# Human Multi-Organoid Platform to Model Immune Dynamics in Cardiac Injury and Disease

**DOI:** 10.1161/CIRCRESAHA.125.326823

**Published:** 2025-09-03

**Authors:** Jasmeet S. Reyat, Yuqi Shen, Gowsihan Poologasundarampillai, Amirpasha Moetazedian, Julie Rayes, Abdullah O. Khan

**Affiliations:** Division of Cardiovascular Medicine, Radcliffe Department of Medicine, John Radcliffe Hospital (J.S.R.), University of Oxford, United Kingdom.; MRC Weatherall Institute of Molecular Medicine, John Radcliffe Hospital (Y.S., A.O.K.), University of Oxford, United Kingdom.; Nuffield Department of Medicine, Ludwig Institute for Cancer Research (A.O.K.), University of Oxford, United Kingdom.; Department of Dentistry, School of Health Sciences (G.P.), University of Oxford, United Kingdom.; Department of Cardiovascular Sciences, School of Medical Sciences, College of Medicine and Health (J.R.), University of Birmingham, United Kingdom.; School of Engineering, Faculty of Science and Engineering, Hull University, United Kingdom (A.M.).; Faculty of Medicine, National Heart and Lung Institute, Hammersmith Hospital, Imperial College London, United Kingdom (J.S.R.).

**Keywords:** fibrosis, heart failure, microphysiological systems, myocytes, cardiac, organoids

**Meet the First Author, see p 1050**

Immune cells play central roles in cardiac injury, repair, and remodeling, ultimately shaping outcomes in conditions such as myocardial infarction, myocarditis, and heart failure. While human in vitro cardiac models have emerged as powerful alternatives to animal models, they have focused on a single, cardiac-only organoid platform that overlooks the recruitment of circulating immune cells and tissue crosstalk.^1^ The lack of tractable human in vitro platforms that model immune recruitment or bone marrow-cardiac crosstalk post-injury is a major bottleneck in the field.

To address this, we present a multi-organoid platform that combines cardiac and bone marrow organoids in a 3D-printed, fluidically connected device housed within a 6-well tissue-culture plate (Figure [Ai]). An incubator rocker drives fluid and cell exchange, mimicking circulation-like dynamics without external pumps (Figure [Aii]). The bone marrow organoid system is derived from our recently published comBO platform,^2^ the first hiPSC (human induced pluripotent stem cell)-derived model to generate a broad spectrum of innate immune cells and adaptive progenitors. We paired this with a vascularized cardiac organoid (VCO) model^3^ to create a highly complex, reproducible, and scalable system for modeling immune-cardiac interactions in ischemic injury (Figure [Ai]). Immune cells are critical players in the resolution of ischemic injury and subsequent remodeling of cardiac tissue that influences long-term outcomes.

**Figure.**
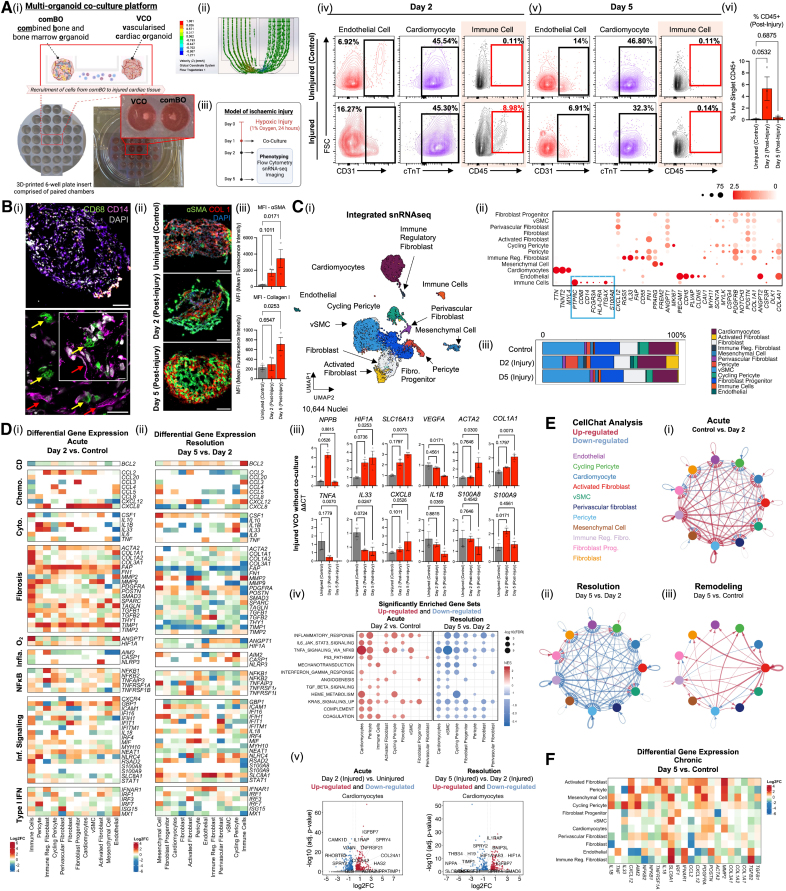
**Design and validation of a cardiac-bone marrow co-culture platform. A**, **i**, Design and implementation of a 3D-printed fluidic device that enables the exchange of cells between a bone marrow organoid (comBO) and vascularized cardiac organoid (VCO) system. **ii**, A fluid flow model depicting the relative flow rate and direction of travel across the fluidic device. **iii**, Ischemic injury was modeled by exposing VCOs to 24 hours of hypoxia (1%) before 5 days of coculture, with phenotyping performed at days 2 and 5 post-injury. **iv** and **v**, Flow cytometry was performed to validate the recruitment of immune cells post-injury. Cells were defined as immune cells (CD45^+^), endothelial cells (CD31^+^ CD45^−^), or cardiomyocytes (cTnT^+^ CD45^−^). A notable CD45^+^ fraction was observed at 2 days post-injury, while no CD45^+^ cells were observed across parallel uninjured controls or at day 5 post-injury. **vi**, CD45^+^ cell recruitment was quantified to confirm a transient recruitment of immune cells at 2 days post-injury. **B**, **i**, Immunofluorescence imaging of cryosections was performed to confirm the recruitment of CD68^+^ macrophages (yellow arrows) and CD14^+^ monocytic cells (red arrows) within the VCO 2 days post-injury (**top**, wide-field overview [inset: 100-µm scale bar]; **bottom**, AiryScan zoomed region (inset: 25-µm scale bar and 10-µm scale bar). **ii**, Similarly, imaging confirmed the onset of fibrosis in VCOs (⍺SMA and collagen type-I) staining, which (**iii**) increased over time (n=3; 1-way ANOVA with multiple comparisons). **C**, **i**, Uniform manifold approximation (UMAP) of single-nucleus snRNA-Seq (single nuclear RNA sequencing) data derived from control (uninjured), day 2, and day 5 VCOs post-injury and coculture. (Total of 10 644 nuclei captured, of which 176 were immune cells.) **ii**, Bubble plot highlighting canonical genes used to annotate the data set. **iii**, Change in distribution of cell types across injury timepoints. **D**, Differential gene expression analysis highlighting the upregulation and downregulation of hypoxic, inflammatory, chemokine, and fibrotic genes at (**i**) day 2 (mimicking acute injury) and (**ii**) day 5 (modeling resolution) post-injury. **iii**, qRT-PCR (quantitative reverse transcriptase polymerase chain reaction) was performed on hypoxia-injured VCOs in the absence of comBO coculturing to determine the distinct effect of coculture on injury (ΔΔCT method calculated relative to uninjured controls). **iv**, Gene set enrichment analysis (GSEA) was performed to identify dysregulation across cell types (FDR=0.3, Wilcox). An upregulation of core inflammatory gene sets was observed at day 2 post-injury, while these gene sets were downregulated at day 5 post-injury, again confirming a model of acute injury and its resolution. **v**, Volcano plots demonstrating gene dysregulation in cardiomyocytes post-injury. **E**, **i**, CellChat analysis of predicted ligand-receptor analysis demonstrates a marked (**ii**) upregulation of cell-cell interactions at day 2 post-injury and (**iii**) subsequent downregulation of these interactions as injury resolves at day 5. **iv**, Analysis of day 5 samples compared with baseline highlights persistent dysregulation consistent with cardiac remodeling post-injury. **F**, Differential gene expression of VCOs at day 5 post-injury highlights a persistent upregulation of hallmark genes reported in cases of poor resolution post-injury, which contribute to remodeling and poor long-term outcomes. **Avi**, **Biii**, and **Diii**: n=3; Kruskal-Wallis tests (with multiple comparisons [Dunn post hoc]) were performed using GraphPad Prism, version 10.3.1. Single-nucleus RNA-seq analysis was performed using R (version 4.4.0). Differential gene expression analysis was performed using the Seurat FindMarkers function (Wilcoxon rank-sum test). Genes with adjusted *P* values (Benjamini-Hochberg) <0.05 and log_2_ fold change >0.25 were identified as differentially expressed. Gene set enrichment was performed using fgsea with the MSigDB Hallmark gene sets. Pathways with FDR-adjusted *P*<0.05 were considered significantly enriched. Note that the low n used in this proof-of-concept study must be taken into account when interpreting statistics. aSMA indicates alpha smooth muscle actin; CD, cell death; Chemo, chemokine; cTnT, cardiac Troponin-T; Cyto, cytokine; FDR, false discovery rate; gsea, gene set enrichment analysis; IFN, interferon; Inflamm, inflammasome; MFI, mean fluorescence intensity; O2, hypoxia; and qRT-PCR, quantitative real time polymerase chain reaction.

To induce ischemia, we subjected VCOs to hypoxia (1% O_2_ for 24 hours), followed by coculture with comBOs within our device for 5 days. VCOs were harvested at days 2 and 5 post-injury for analyses by flow cytometry, immunofluorescence, and single nucleus RNA sequencing (snRNA-Seq) (Figure [Aiii]).

Flow cytometry demonstrated a recruitment of CD45^+^ immune cells on day 2, which returned to baseline by day 5 (Figure [Aiv and Av]). No CD45^+^ immune cells were found in uninjured VCOs, confirming injury-specific recruitment (Figure [Avi]). Immunofluorescence imaging of cryosections collected at day 2 post-injury confirmed the presence of CD14^+^ monocytic cells and CD68^+^ macrophages within the organoid volume (Figure [Bi]). Imaging also confirmed the onset of fibrosis post-injury, evidenced by an increase in αSMA and collagen type-1 deposition (Figure [Bii]).

snRNA-Seq identified major myocardial cell types: cardiomyocytes (*TTN*, *TNNT2*, and *MYL4*), fibroblast subsets (*PDGFRB* and *POSTN*) including immune-regulatory fibroblasts (*IL33*, *CD55*, and *RGS5*), pericytes (*CSPG4* and *PDGFRB*), microvascular endothelial cells (*PECAM1*, *CDH5*, continuous [*CLDN5/CAV1*], and fenestrated [*PLVAP*] subsets), and infiltrating immune cells (*PTPRC/CD45*, *CD68*, and *CD14*; Figure [Ci and Cii]). The immune cluster included myelomonocytic cells (*CD68* and *CD14*), granulocytes (*FCGR3A*), dendritic cells (*ITGAX* and *CD11c*), and neutrophils (*S100A8*) though the total number of immune nuclei captured was insufficient for a detailed subclustering of this compartment. Notably, activated fibroblasts increased and vascular smooth muscle cells declined in number at day 2 post-injury (Figure [Ciii]).

Differential expression analysis at day 2 post-injury revealed an upregulation of hypoxia-responsive (*HIF1A* and *ANGPT1*), fibrotic (*TGFB1*, *COL1A1*, *MMP9*, and *TIMP1*), inflammatory (*IL1B* and *NFKB1*), and chemokine genes (*CXCL8*, *CCL2*, and *CCL3*; Figure [Di]). By day 5, there was a downregulation of fibrotic (*MMP9*, *ACTA2*, *THGB1*, and *COL1A1*) and inflammatory (*MX1*, *IRF1*, *IRF7*, *S100A8*, *IL6*, *TNF*, and *IL10*) genes, indicating a resolution of the acute injury observed at day 2 (Figure [Dii]). qRT-PCR (quantitative reverse transcriptase polymerase chain reaction) performed on injured VCOs without comBO coculture confirmed the importance of immune cell recruitment in the induction of inflammation (Figure [Diii]). Future work will directly compare monoculture and coculture with snRNA-Seq to identify immune-dependent pathways.

Gene set enrichment analysis confirms the upregulation (day 2 post-injury) and downregulation (day 5 post-injury) of hallmark pathways associated with ischemic injury,^4^ confirming a dynamic model of acute injury and its resolution (Figure [Div]).

Cardiomyocyte-specific differentially expressed genes (DEGs) included genes implicated in human ischemic disease (*IGFBP7*, *HAS2*, *TIMP1*, *IL1RAP*, and *HIF1A*^5^; Figure [Dv]). CellChat analysis of ligand-receptor interactions revealed increased cell-cell interactions at day 2 and a pronounced decline by day 5 (Figure [Ei and Eii]), consistent with other analyses^5^ and the current model of the resolution of ischemic injury. Interestingly, a comparison of day 5 data to baseline (Figure [Eiii]) indicates that a degree of dysregulation persists, consistent with remodeling post-injury. This was confirmed in DEG analysis comparing day 5 data to baseline, which highlighted markers of fibroinflammatory remodeling following cardiac injury (*IL18*, *CSF1*, *MMP2*, and *SLC8A*); these features correlated to poor long-term outcomes.^5^

Together, these findings establish a scalable human in vitro platform that captures the dynamic interplay between immune recruitment, inflammatory resolution, and tissue remodeling following ischemic cardiac injury. We demonstrate a clear temporal progression: an acute phase marked by immune cell infiltration and proinflammatory transcriptional activation at day 2, transitioning to a resolution phase by day 5, and finally hallmarks of fibroinflammatory remodeling.

This platform addresses a critical need by enabling human-specific studies of immune-driven cardiac injury, repair, and remodeling. We offer a viable alternative to animal models, opening new avenues for mechanistic discovery and therapeutic testing in human tissue, with direct relevance to ischemic disease and heart failure. This approach can be easily adapted to include new and emerging organoid models, including a broader repertoire of immune-relevant tissues that contribute to cardiovascular pathology.

## Article Information

### Acknowledgments

The authors thank the MRC Weatherall Institute of Molecular Medicine Genomics and Flow Cytometry facilities for their help with preparing sequencing libraries and running nuclei sorting, respectively. In addition, the authors thank Dr Michela Noseda (National Heart and Lung Institute, Imperial College London) for providing protocols for nuclei isolation.

### Sources of Funding

This study was supported by a University of Oxford John Fell Fund Award 0014223 and a Sir Henry Wellcome Fellowship (grant 218649/A/19/Z) awarded to A.O. Khan. J.S. Reyat was supported by a British Heart Foundation Chair Award to Professor Barbara Casadei (grant CH/SV/22/280022). J. Rayes is a British Heart Foundation Intermediate Fellow (grant FS/IBSRF/20/25039).

### Disclosures

A patent has been filed in reference to this work (GB2507997.1). All the authors report no other relevant disclosures. Sequencing data and an annotated R object are available upon publication through the Gene Expression Omnibus (GEO) accession number: GSE305006. Experimental methods will be made available through our online group (https://groups.google.com/g/moreganoids) upon publication.

## Supplementary Material


