# Supplementary material for: Human Multi-Organoid Platform to Model Immune Dynamics in Cardiac Injury and Disease
Source: Circ Res. 2025 Sep 3;137(8):1133–6. doi: 10.1161/CIRCRESAHA.125.326823 (PMC12466161; doi:10.1161/CIRCRESAHA.125.326823)
Supplement: Supplementary file 1 [file res-137-1133-s001.pdf]

Major Resources Table

In order to allow validation and replication of experiments, all essential research materials listed in the Methods should be included in the Major Resources Table below. Authors are encouraged to use public repositories for protocols, data, code, and other materials and provide persistent identifiers and/or links to repositories when available. Authors may add or delete rows as needed.

Animals (in vivo studies)

Species	Vendor or Source	Background Strain	Sex	Persistent ID / URL
N/A				

Genetically Modified Animals

	Species	Vendor or Source	Background Strain	Other Information	Persistent ID / URL
**Parent - Male**	N/A				
**Parent - Female**	N/A				

Antibodies

Target antigen	Vendor or Source	Catalog #	Working concentration	Lot # (preferred but not required)	Persistent ID / URL
CD45, FITC	Biolegend	368508	1:100		RRID:AB_2566368
CD31, APC-Cy7	Biolegend	303120	1:100		RRID:AB_10640734
CD90, APC	Biolegend	328114	1:100		RRID:AB_893431
cTnT, PE	Miltenyi Biotec, REA400 1C11	130-120-545	1:50		RRID:AB_2801770
αSMA	Sigma Aldrich, 1A4	A2547	1:500	0000202620	RRID:AB_476701
Collagen I	Sigma Aldrich, COL-1	C2456	1:500	0000365056	RRID:AB_476836
CD68	eBioscience, KP1	14-0688-82	1:500	2923549	RRID:AB_11151139
CD14	Abcam, SP192	ab183322	1:500	GR3367445-7	RRID:AB_2909463
Alexa Fluor 488 goat anti-mouse IgG2a (y2a)	ThermoFisher	A21131	1:200	2836752	RRID:AB_141618
Alexa Fluor 568 goat anti-mouse IgG1 (y1)	ThermoFisher	A21124	1:200	2997081	RRID:AB_141611
Alexa Fluor 674 goat-anti-mouse IgG (H+L) Cross-Adsorbed	ThermoFisher	A21445	1:200		RRID:AB_2535862
Alexa Fluor 488 goat anti-rabbit IgG (H+L) Cross-Absorbed	ThermoFisher	A11008	1:200		RRID:AB_143165

DOI [to be added]

DNA/cDNA Clones

Clone Name	Sequence	Source / Repository	Persistent ID / URL
N/A			

Cultured Cells

Name	Vendor or Source	Sex (F, M, or unknown)	Persistent ID / URL
Gibco^{TM} episomal hiPSC line, A18945	Gibco	F	https://hpscreg.eu/cell-line/TMOi001-A

Data & Code Availability

Description	Source / Repository	Persistent ID / URL
Single nuclear RNA Sequencing	NCBI Gene Expression Omnibus	GEO: GSE305006
Experimental methods	Moreganoids Google Group	https://groups.google.com/g/moreganoids

Other

Description	Source / Repository	Persistent ID / URL

DOI [to be added]

ARRIVE GUIDELINES

The ARRIVE guidelines (https://arriveguidelines.org/) are a checklist of recommendations to improve the reporting of research involving animals. Key elements of the study design should be included below to better enable readers to scrutinize the research adequately, evaluate its methodological rigor, and reproduce the methods or findings.

Study Design

**Groups**	**Sex**	**Age**	**Number (prior to experiment)**	**Number (after termination)**	**Littermates (Yes/No)**	**Other description**
Group 1 (Control)						
Group 2						
Add more if needed						

Sample Size: Please explain how the sample size was decided Please provide details of any a *prior* sample size calculation, if done.

Inclusion Criteria

Exclusion Criteria

Randomization

Blinding

DOI [to be added]
